# Identification of a factor controlling lysosomal homeostasis using a novel lysosomal trafficking probe

**DOI:** 10.1038/s41598-019-48131-2

**Published:** 2019-08-12

**Authors:** Shunsuke Ishii, Akira Matsuura, Eisuke Itakura

**Affiliations:** 1 Department of Biology, Graduate school of Science and Engineering, Chiba University, Inage-ku, Chiba, 263-8555 Japan; 2 Department of Biology, Graduate school of Science, Chiba University, Inage-ku, Chiba, 263-8555 Japan

**Keywords:** Lysosomes, Lysosomes

## Abstract

Lysosomes are largely responsible for significant degradation of intracellular and extracellular proteins via the secretory pathway, autophagy, and endocytosis. Therefore, dysregulation of lysosomal homeostasis influences diverse cellular functions. However, a straightforward and quantitative method to measure the integrity of the lysosomal pathway has not been developed. Here, we report the plasmid-based lysosomal-METRIQ (MEasurement of protein Transporting integrity by RatIo Quantification) probe that enables simple quantification of lysosomal integrity by lysosomal green and cytosolic red fluorescent proteins using a flow cytometer. In cultured cells, the lysosomal-METRIQ probe detected not only suppression of the lysosomal pathway but also upregulation of lysosomal activity such as lysosomal biogenesis. To identify factors involved in lysosomal homeostasis, we carried out compound screening and found that the cyclin-dependent kinase (CDK) inhibitors kenpaullone and purvalanol A induce synthesis of cathepsin D and an increase in the number of lysosomes. Subsequent studies revealed that CDK5 maintains lysosomal homeostasis independently of cell cycle arrest. Our results suggest that the lysosomal-METRIQ probe is an effective and efficient tool for measuring lysosomal activity in mammalian cells.

## Introduction

Eukaryotic cells have an endomembrane system responsible for the biosynthesis and transport of diverse macromolecules among membrane-bound compartments involved in degradative, endocytic, and secretory processes. Although the organelles, such as the endoplasmic reticulum (ER), Golgi apparatus, endosomes, and lysosomes, have different functions and properties, protein and lipid components are dynamically exchanged among them^1,2^. Continuous exchange among compartments maintains organelle populations. However, each organelle exhibits responses to particular stresses or cell cycle conditions.

Lysosomes are membrane-bound intracellular organelles that play important roles in the degradation of macromolecules including proteins, lipids, polysaccharides, and nucleic acids from both external and internal origins. Lysosomal substrates are transported from several locations including the ER, plasma membrane, extracellular compartment, and cytosol via the secretory, endocytic, and autophagic pathways^3,4^. Substrates in the lysosomal lumen, which is maintained at an acidic internal pH by an ATP-dependent proton pump, are degraded by over 60 hydrolases consisting of proteases, glycosidases, lipases, phosphatases, sulphatases, and nucleases^5^. Lysosomes are the terminal degradative compartment, and the resulting catabolites are exported to the cytosol and reused in cellular metabolism.

Recent works have revealed that the lysosome also plays critical roles in nutrient sensing and signalling hubs involved in cell growth and metabolism^3,6^. When nutrients are sufficient, insulin and amino acids activate mammalian target of rapamycin complex 1 (mTORC1) at the lysosome^7,8^. Activated mTORC1 phosphorylates translation factors such as ribosomal protein S6 and eukaryotic translation initiation factor 4E-binding protein (4E-BP) 1, which induce cell growth. Transcription factor EB (TFEB) is also phosphorylated by mTORC1 and sequestered in the cytosol via binding with 14-3-3 proteins. Upon nutrient starvation, suppression of mTORC1 results in decreases in the phosphorylation of substrates, including TFEB. Dephosphorylated TFEB is released from binding to 14-3-3 and is translocated from the cytosol to the nucleus to activate the expression of a unique set of genes related to lysosomal biogenesis^9–11^. Thus, the lysosomal lumen and membrane play multiple roles related to digestion, metabolite transport, signalling hub, trafficking, lumen acidification, and fusion with other organelles. Therefore, a defect in a lysosomal pathway due to a single mutation has secondary effects on other lysosomal pathways. Indeed, lysosome dysfunction due to genetic defects in lysosomal proteins causes lysosomal storage diseases and acquired metabolic disorders, of which more than 40 are known^12,13^.

Quantifying lysosomal activity is important for cell biological studies. To date, several methods have been used to study lysosomal homeostasis. For example, LysoTracker dyes, which are selectively incorporated in vesicles with low pH, are used as pH reporters for lysosomes^14^. Self-quenching fluorescence-labelled proteins that produce fluorescence upon cleavage by lysosomal proteases after endocytosis are also used^15^. Immunostaining of lysosomal membrane proteins can be used to assess lysosomal numbers and morphology^16,17^. The classical methods for detecting lysosomal protease activity are the cathepsin maturation assay, which is performed by a pulse-chase approach using radioisotopes, or immunoblotting using anti-cathepsin antibodies^18^. Although these methods are widely used, they have trade-offs associated with radioisotope contamination and cost. In addition, intensity-based fluorescent probes are prone to errors associated with cell densities, probe concentration, and incorporation rate^19^. We thus sought to develop a plasmid-based lysosomal ratiometric fluorescent probe to provide a simple quantitative method for measuring lysosomal activity. In addition, it was essential that the technique be simple and inexpensive.

In this study, we characterised a novel lysosomal probe that detects lysosomal protease activity based on red/green fluorescence signal ratios in single living cells. The probe also monitors the homeostasis of the membrane trafficking pathway from the ER to lysosomes, as well as lysosomal biogenesis. Using this probe, we conducted a comprehensive analysis of the lysosomal pathway in cells treated with 368 different drugs, and we here show that the probe can be successfully employed to identify undiscovered compounds affecting lysosomal function. We confirmed that cyclin-dependent kinase 5 (CDK5) is involved in lysosomal activity. Our data suggest that CDK5 maintains lysosomal homeostasis by regulating lysosomal biogenesis.

## Results

### Development of the lysosomal-METRIQ probe, a novel fluorescent construct

**Figure 1:**
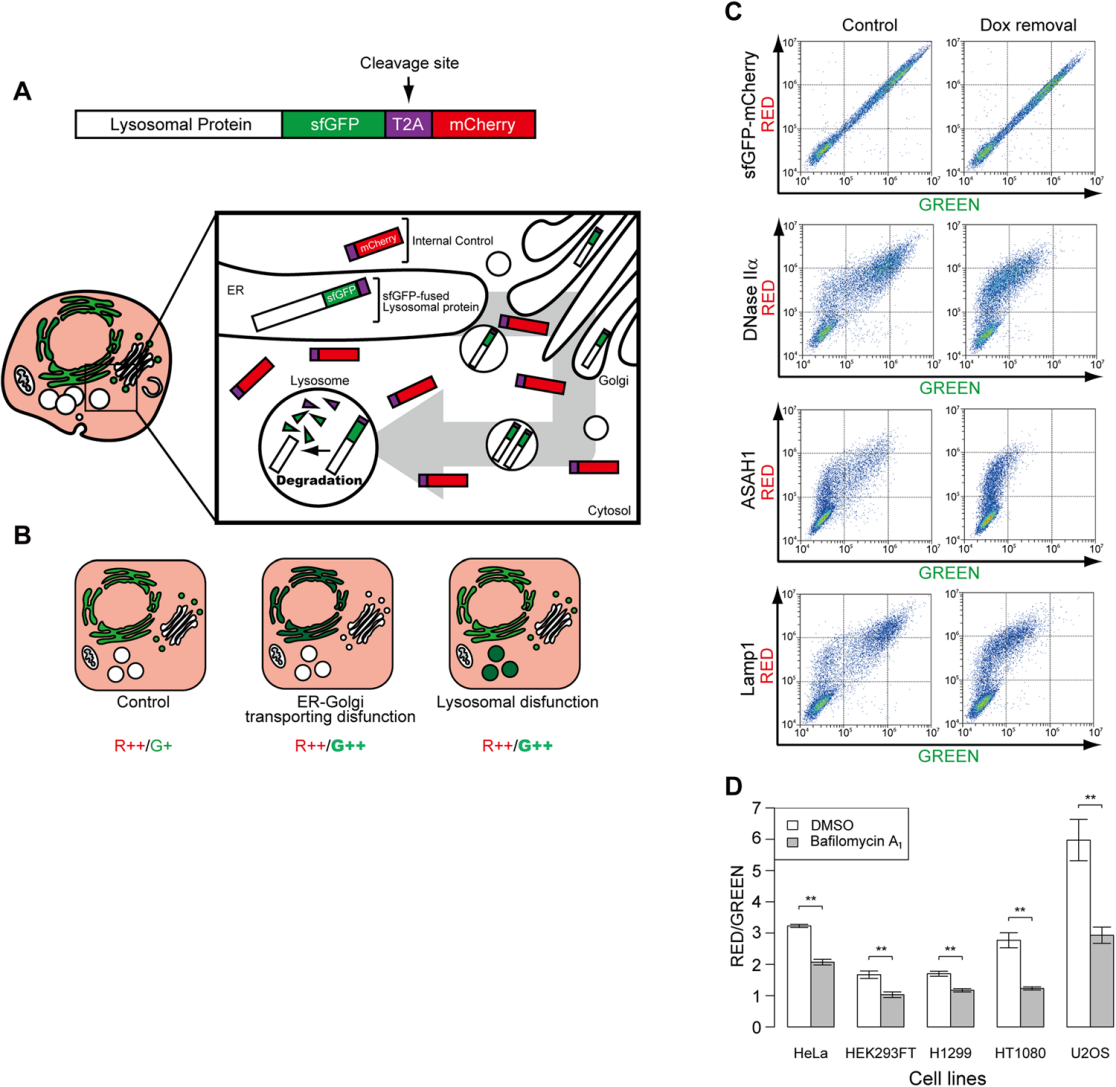
Development of the lysosomal-METRIQ probe, a novel fluorescent construct. (**A**) Schematic diagrams of the measurement of membrane trafficking and lysosome integrity using fluorescent proteins. (**B**) Predicted phenotypes of the lysosomal-METRIQ probe against several stresses. (**C**) sfGFP, but not mCherry, is degraded in lysosomes. Tetracycline-On (Tet-On) HeLa cells expressing sfGFP-mCherry as a negative control or the lysosomal-METRIQ probe containing DNase IIα, ASAH1, or LAMP1 were cultured in the presence of doxycycline (Dox). Following removal of Dox, the cells were incubated for 12 h prior to flow cytometry. Dot plots of GFP versus mCherry fluorescence intensities are shown. (**D**) Differences in the lysosomal pathway or lysosomal activity under basal conditions among the cell lines. HeLa, HEK293FT, H1299, HT1080, and U2OS Tet-On cells expressing the lysosomal-METRIQ probe were cultured with Dox for 48 h and treated with vehicle (DMSO) or bafilomycin A_1_ for 12 h prior to flow cytometry (n = 3). The data represent the mean ± standard deviation (SD); **p < 0.01.

A versatile and straightforward method to quantify the integrity of the endomembrane trafficking pathway from the ER to lysosomes has not yet been developed. To generate a simple and quantitative approach for detecting lysosomal integrity, we constructed a novel probe, the lysosomal-METRIQ (MEasurement of protein Transporting integrity by RatIo Quantification) probe that expresses a fusion protein consisting of a lysosomal protein linked with superfolder green fluorescent protein (sfGFP), a self-cleaving T2A peptide, and mCherry^20^. During translation of the lysosomal-sfGFP-T2A-mCherry protein, the signal peptide of the nascent chain is recognised by the signal recognition particle to enable insertion into the translocon on the ER membrane. Meanwhile, the nascent chain is cleaved at the T2A sequence, and the C-terminal mCherry is released into the cytosol. Therefore, the total intensity of mCherry (red fluorescence) can be used as an internal control. When the membrane trafficking pathway from the ER to lysosomes is intact, the lysosomal sfGFP protein, but not mCherry, is transported into lysosomes. sfGFP loses its fluorescence in acidic conditions and is immediately degraded by lysosomal proteases^21^ (Fig. 1A). In contrast, under conditions of disturbed membrane trafficking or lysosomal activity, sfGFP accumulates. This novel probe can be used to analyse the integrity of the membrane trafficking pathway between ER and lysosomes through examination of the ratio of fluorescence intensity (RED/GREEN; R/G). Theoretically, the localisation and intensity of sfGFP fluorescence should fluctuate upon disturbance of the endomembrane trafficking pathway, while the mCherry should remain stable in the cytosol and nucleosol. Therefore, we can readily observe dysfunction of the endomembrane system by microscopy (Fig. 1B). To target sfGFP to lysosomes, three lysosomal proteins, deoxyribonuclease (DNase IIα), acid ceramidase (ASAH1), and lysosomal-associated membrane protein 1 (LAMP1; a lysosomal membrane protein), were inserted into a vector containing sfGFP-T2A-mCherry with the Tet-On system. First, we assessed whether the lysosomal sfGFP proteins in the probe were degraded efficiently by measuring sfGFP fluorescence intensity using a flow cytometer. Following doxycycline (Dox) induction, cells were analysed either under steady-state conditions (Fig. 1C, left panels), or after a 12 h chase (right panels) to allow for degradation of sfGFP when preceded by a lysosomal targeting signal. Rapid decay of sfGFP fluorescence was observed by Dox removal in all lysosomal proteins tested, while the fluorescence of sfGFP-mCherry alone did not decrease (Fig. 1C). These data suggest that lysosomal sfGFP proteins in the METRIQ probe are immediately degraded in lysosomes. Furthermore, mCherry is stable even after Dox removal. Distinct differences in sfGFP degradation rates were not observed among the different lysosomal proteins. We ultimately chose DNase IIα for the lysosomal-METRIQ probe because its deoxyribonuclease activity should have a minimal effect on cellular protein degradation activity. We also characterised lysosomal activities in other cell lines (HeLa, HEK293FT, H1299, HT1080, and U2OS) using the lysosomal-METRIQ probe. These cell lines showed distinct differences in lysosomal activity, and bafilomycin A_1_ treatment significantly decreased the R/G ratio in all cell lines tested (Fig. 1D). These data suggest that the lysosomal-METRIQ probe can be used as an indicator for the integrity of the lysosomal pathway regardless of cell type.

### The lysosomal-METRIQ probe detects lysosomal activity

Newly synthesised lysosomal proteins in the ER are transported to the Golgi apparatus and are then distributed to lysosomes. We examined the sensitivity of the lysosomal-METRIQ probe to disturbance of the lysosomal transport pathway in mammalian cells.

**Figure 2:**
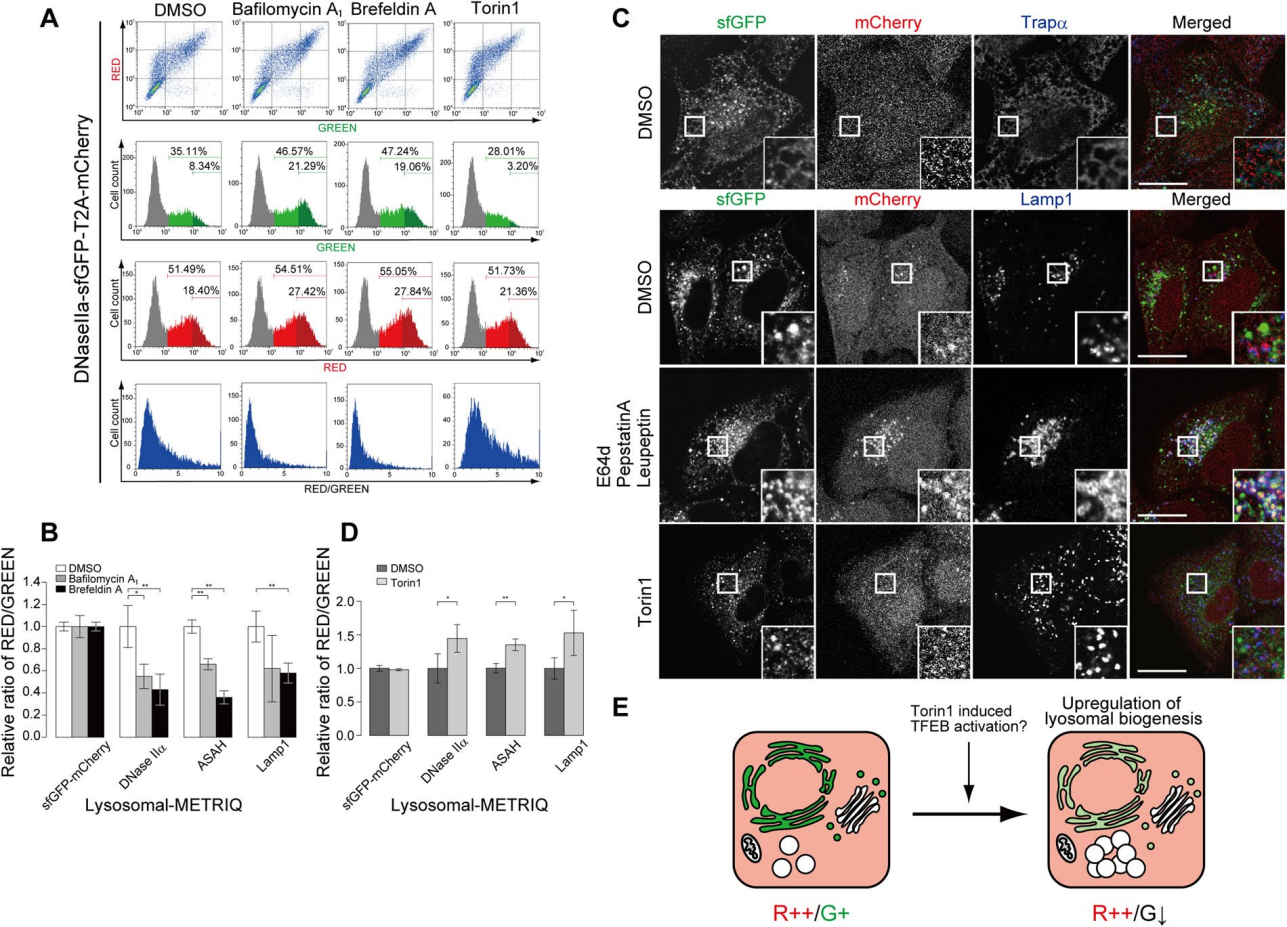
The lysosomal-METRIQ probe detects lysosomal integrity. (**A**) Detection of several stresses induced by representative inhibitors using flow cytometry. HeLa Tet-On cells expressing the lysosomal-METRIQ probe were cultured with Dox for 48 h and treated with vehicle (DMSO), bafilomycin A_1_, brefeldin A, or Torin1 for 12 h prior to analysis. The green and red fluorescence intensities were determined by flow cytometry. Representative dot plots of sfGFP versus mCherry fluorescence intensity and corresponding histograms are shown. (**B**) The red/green fluorescence ratios are shown (n = 4). The fluorescence ratios were based on the high green emission window as defined in (**A**). The data represent the mean ± SD. The data are representative of at least three independent experiments; *p < 0.05 and **p < 0.01. (**C**) DNase IIα-sfGFP, but not mCherry, is transported to lysosomes. HeLa cells expressing the lysosomal-METRIQ probe were incubated in medium containing Dox for 48 h and treated with vehicle (DMSO), E64d with pepstatin Aand leupeptin, or Torin1 for 24 h before fixing. Cells were stained with antibodies against LAMP1 and TRAPα, and analysed by immunofluorescence microscopy. A magnified image of the indicated region is shown in the inset. Scale bar, 20 μm. (**D**) The lysosomal-METRIQ probe can also detect increased lysosomal biogenesis. HeLa cells expressing the probe were cultured with Dox for 48 h and treated with the mTOR inhibitor Torin1 for 12 h prior to flow cytometry. The red/green fluorescence ratios are shown (n = 4). The data represent the mean ± SD; *p < 0.05 and **p < 0.01. (**E**) Phenotype of the lysosomal-METRIQ probe under lysosomal upregulation.

HeLa Tet-On cells expressing the lysosomal-METRIQ probe were treated with bafilomycin A_1_, an agent that selectively inhibits vacuolar proton ATPase leading to lysosomal neutralisation and membrane trafficking dysfunction, or with brefeldin A, an agent that disrupts protein transport from the ER to the Golgi, for 12 h before analysis by flow cytometry. Treatment with bafilomycin A_1_ caused significant accumulation of GFP fluorescence, while mCherry fluorescence intensity was not markedly changed (Fig. 2A). As a result, the R/G ratio of the lysosomal-METRIQ probe, but not sfGFP-mCherry, was two-fold lower in bafilomycin A_1_ treated cell than in non-treated cells (Fig. 2B). Brefeldin A treatment also caused a similar reduction in the R/G ratio. These data suggest that the lysosomal-METRIQ probe can be used to measure disturbances in both the transport pathway towards lysosomes and lysosomal activity based on fluorescence ratios.

We next verified the localisation of the probe in cells to confirm the lysosomal transport of DNase IIα-sfGFP. HeLa cells expressing the lysosomal-METRIQ probe in the presence or absence of lysosomal inhibitor were fixed, stained with antibodies against endomembrane organelles, and examined by microscopy. In regular medium, DNase IIα-sfGFP showed reticular and punctate structures and did not co-localized with GM130 (a Golgi marker), TMP21 (a Golgi marker) or EEA1 (an early endosome marker) (Fig. S1), whereas it did co-localise with translocon-associated protein (TRAP) subunit alpha (TRAPα; an ER marker) (Fig. 2C). These punctate structures rarely colocalised with LAMP1 (lysosome marker)-positive structures. In contrast, following inactivation of lysosomal proteases with a lysosomal inhibitor cocktail containing E64d, pepstatin A, and leupeptin, punctate structures of sfGFP signals were increased and were clearly colocalised with LAMP1-positive structures (Fig. 2C). These data suggest that DNase IIα-sfGFP is successfully transported to lysosomes and rapidly degraded by lysosomal proteases.

Lysosomal biogenesis is known to be regulated by TFEB, a master regulator that activates lysosomal genes. Thus, we examined whether the lysosomal-METRIQ probe is sensitive to enhanced lysosomal biogenesis. We treated cells expressing the probe with the mTOR inhibitor Torin1 to activate TFEB to promote lysosomal biogenesis. The sfGFP signal, but not the mCherry signal, was decreased two-fold compared to no treatment with Torin1 treatment (Fig. 2C,D). Fluorescence microscopy revealed a decrease in sfGFP intensity, while the localisation of the probe was not affected. These data suggest that the lysosomal-METRIQ probe can be used to monitor lysosomal homeostasis such as lysosomal dysfunction and enhanced biogenesis (Figs 1B, 2E).

### Compound screening of the lysosomal pathway with the lysosomal-METRIQ probe

**Figure 3:**
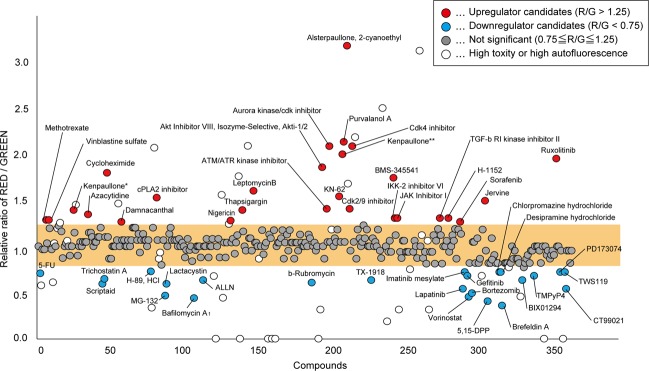
Drug screening of the lysosomal pathway by the lysosomal-METRIQ probe. Evaluation of the effects of known cancer-related inhibitors against the membrane-trafficking pathway and lysosomal activity. HeLa Tet-On cells expressing the lysosomal-METRIQ probe were cultured in regular medium with Dox for 48 h and treated with each drug from the SCADS inhibitor library for 12 h prior to analysis. The red/green fluorescence ratio was measured using flow cytometry. Kenpaullone obtained from ALEXIS and Calbio chem is indicated with * and **, respectively. Candidate membrane trafficking or lysosomal activity downregulators are coloured in blue. Candidate lysosomal biogenesis upregulators are coloured in red.

Accumulating evidence indicates that lysosomes are regulated by intracellular signalling pathways^6^. To identify a novel signalling regulator or regulators in the lysosomal pathway, we used a Screening Committee of Anticancer Drugs (SCADS) inhibitor kit that contained 368 types of tumour-related inhibitors, including many compounds affecting intracellular signalling pathways, using cells expressing the lysosomal-METRIQ probe. HeLa cells expressing the lysosomal-METRIQ probe were treated with each compound (10 μM) for 12 h, and the R/G ratios were measured by flow cytometry (Fig. 3 and Table S1). We excluded compounds that caused a cell death rate of greater than 50% and compounds with high auto-fluorescence. Based on the screening results, 25 drugs caused a > 25% decrease in the R/G ratio (downregulator candidates), while 26 drugs induced a > 25% increase in the R/G ratio (upregulator candidates).

Among the 25 downregulator candidates, the ATPase inhibitor bafilomycin A_1_ and the ER to Golgi transport inhibitor brefeldin A profoundly decreased the R/G ratio, as expected. Inhibitors of proteasome, telomerase, and methylation also significantly reduced the ratio.

Among the 26 upregulator candidates, the chromosomal region maintenance 1 (CRM1) inhibitor leptomycin B mildly increased the ratio, possibly through the same mechanism as the mTOR inhibitor Torin1, inducing localisation of TFEB (a transcription factor that regulates lysosomal biogenesis) to the nucleus^9,22^. On the other hand, treatment with inhibitors of CDKs, hedgehog, and IKKβ, which have not been reported to be regulatory components of lysosomal biogenesis, notably increased the R/G ratio. These results might suggest that the transport pathway from the ER to lysosomes, as well as lysosomal biogenesis itself, may be regulated by several distinct molecular mechanisms.

### Involvement of IKKβ and hedgehog signalling inhibitors in lysosomal function

Our screening indicated that 26 types of compounds out of 368 drugs increased the R/G ratio of the lysosomal-METRIQ probe by 1.25-fold or greater. Among these, several compounds that target the hedgehog or IKK signalling pathways were found as lysosomal upregulators. We next explored how the compounds induced lysosomal biogenesis.

The results of repeated flow cytometry experiments using jervine, an agent that selectively inhibits Smoothened under hedgehog signalling, and BMS-345541, an inhibitor of IKKβ, showed that these compounds increased the R/G ratio (Fig. S2A)^23^. Since nuclear translocation of TFEB activates lysosomal activity, we generated HeLa cells stably expressing TFEB-sfGFP, and examined whether these compounds promoted nuclear translocation of TFEB. The mTOR inhibitor Torin1 clearly induced the nuclear translocation of TFEB (Fig. S2B). Unexpectedly, treatment with jervine for 12 h did not promote nuclear localisation of TFEB, while half of the cell population treated with BMS-345541 exhibited forced translocation of TFEB (Fig. S2B,C). Additionally, the phosphorylation levels of TFEB decreased in a time-dependent manner following treatment with BMS-345541 (Fig. S2D). These data suggest that two different types of lysosomal activation may exist.

To validate the direct effect of IKKβ in lysosomal activation, we attempted to suppress IKKβ by knocking down IKKβ protein using small interfering RNA (siRNA) targeting IKKβ or through overexpression of a dominant-negative mutation, IKKβ K44A^24^. Unlike treatment with BMS-345541, nuclear localisation of TFEB was not observed (Fig. S2E,F), and the R/G ratio of the lysosomal-METRIQ probe did not change with siRNA (Fig. S2F,G). As BMS-345541 treatment did not involve phosphorylation of downstream factors of mTOR (Fig. S2D), it is unlikely that BMS-345541 regulates TFEB activity via mTOR. Therefore, these data suggest that BMS-345541 induces lysosomal activation independently of IKKβ.

We also tested whether Smoothened, a target of jervine, functions directly in upregulation of lysosomal activity. However, lysosomal upregulation was not observed in cells with siRNA targeting Smoothened (Fig. S2F). These data suggest that jervine regulates lysosomal function via an unknown pathway independently of Smoothened.

### CDK5 mediates repression of the lysosomal biogenesis pathway, independently of TFEB

Our screening revealed that several CDK inhibitors including kenpaullone, purvalanol A, alsterpaullone, CDK2/9 inhibitor, and CDK4 inhibitor, dissipated sfGFP signal, suggesting that CDK activity may regulate lysosomal biogenesis (Fig. 3 and Table S1). There are two possible mechanisms: (1) cell cycle arrest itself induces lysosomal biogenesis, or (2) CDK directly modulates lysosomal biogenesis independently of cell cycle arrest.

**Figure 4:**
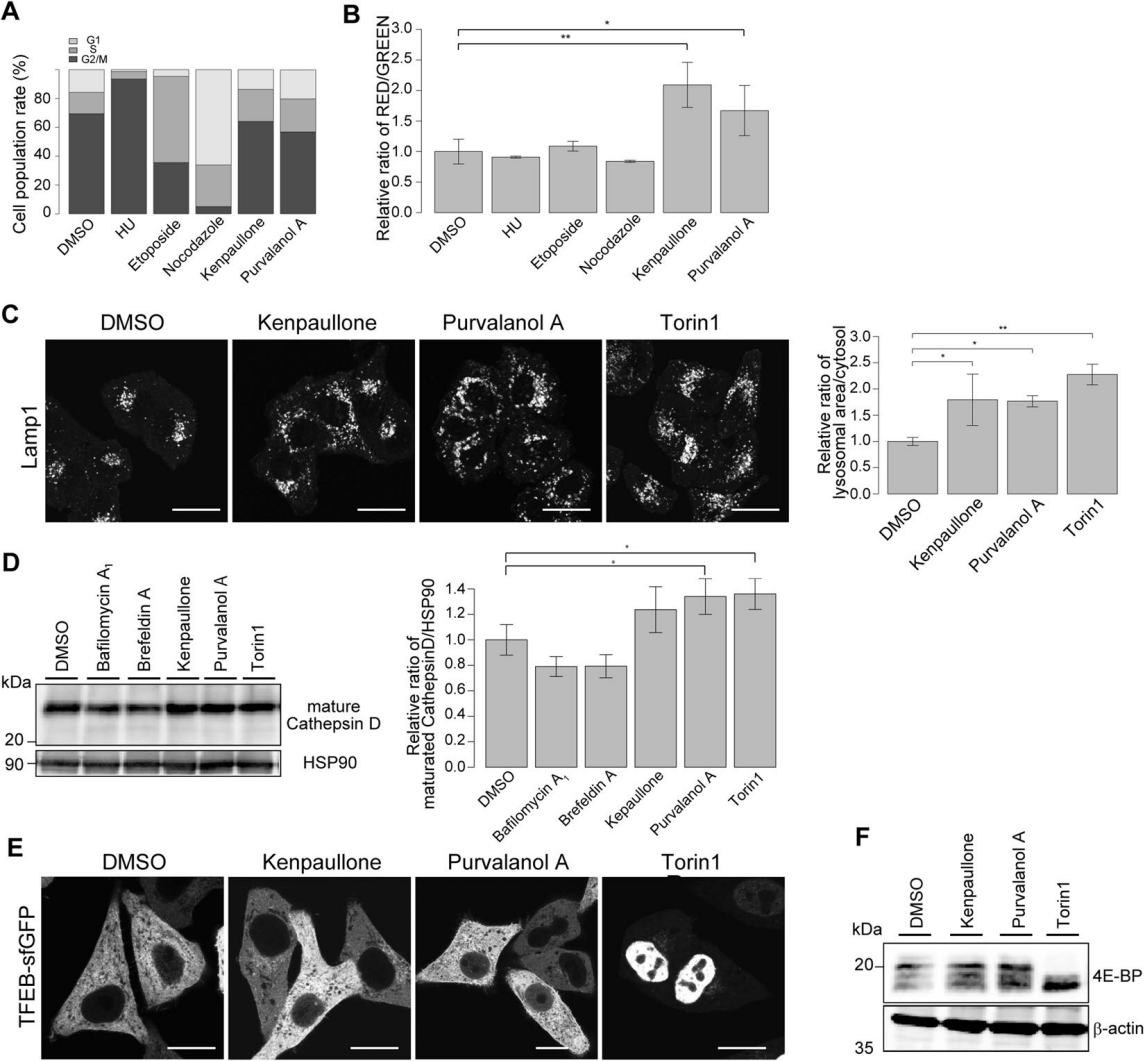
Treatment of kenpaullone or purvalanol A leads in lysosomal biogenesis, independently of cell cycle arrest. (**A**) Treatment with major CDK inhibitors does not increase cell populations belonging to a particular phase of the cell cycle. HeLa cells were treated with vehicle (DMSO), hydroxyurea (HU), etoposide, nocodazole, kenpaullone, or purvalanol A for 12 h before fixation. Following RNase treatment, cells were stained with propidium iodide and fluorescence intensity was measured by flow cytometry. The percentage of cells in each phase is shown by a stacked bar graph. (**B**) Major cell cycle inhibitors do not change the red/green ratios of the lysosomal-METRIQ probe. HeLa Tet-On cells expressing the lysosomal-METRIQ probe were cultured with Dox for 48 h followed by treatment with vehicle (DMSO), HU, etoposide, nocodazole, kenpaullone, or purvalanol A for 12 h prior to analysis. The green and red fluorescence intensities were determined by flow cytometry. The red/green fluorescence ratios are shown. The data represent the mean ± SD; *p < 0.05 and **p < 0.01. (**C**) Treatment with kenpaullone or purvalanol A significantly increases LAMP1-positive structures. HeLa cells were treated with vehicle (DMSO), kenpaullone, purvalanol A, or Torin1 for 12 h before fixation. The cells were stained with antibodies against LAMP1 and analysed by immunofluorescence microscopy. Scale bar, 20 μm. The graph shows the relative ratios of the LAMP1-positive structure areas to the cytoplasmic areas of the cells treated with the indicated compounds (n = 3). The data represent the mean ± SD; *p < 0.05 and **p < 0.01. (**D**) Endogenous mature cathepsin D in HeLa cells treated with vehicle (DMSO), bafilomycin A_1_, brefeldin A, Torin1, kenpaullone, or purvalanol A was analysed by immunoblotting using antibodies against cathepsin D and Hsp90. The mature cathepsin D expression levels were normalised to the HSP90 expression levels (right panel). The data represent the mean ± SD; *p < 0.05. (**E**) Inhibition of CDKs does not induce nuclear localisation of TFEB. HeLa cells stably expressing TFEB-sfGFP were treated with vehicle (DMSO), kenpaullone, purvalanol A, or Torin1 before fixation and were analysed by immunofluorescence microscopy. Scale bar, 20 μm. (**F**) Inhibition of CDKs does not decrease mTOR activity. Endogenous phosphorylated 4E-BP in HeLa cells treated with Torin1, kenpaullone, or purvalanol A was analysed by immunoblotting using antibodies against 4E-BP and β-actin (loading control).

Because the sequence similarity among CDK family proteins is very high, it is difficult to inhibit only one specific CDK protein using an inhibitor^25^. Kenpaullone and purvalanol A are known to act on multiple CDK proteins^26,27^. We therefore determined whether treatment with kenpaullone or purvalanol A affects the HeLa cell cycle by propidium iodide staining. Treatment with kenpaullone or purvalanol A did not increase the proportion of cells in any specific cell cycle phase, unlike hydroxyurea (HU), etoposide, or nocodazole treatment (Fig. 4A), suggesting that kenpaullone and purvalanol A can induce cell cycle arrest at any phase. Importantly, cell cycle arrest caused by HU, etoposide or nocodazole treatment did not affect lysosomal activity measured with the lysosomal-METRIQ probe (Fig. 4B). These data suggest that upregulation of lysosomal activity by treatment with kenpaullone or purvalanol A occurs independently of cell cycle arrest.

We verified that treatment with kenpaullone or purvalanol A increased the R/G ratios in cells expressing the lysosomal-METRIQ probe (Fig. 4B). To confirm whether the increase of the lysosomal-METRIQ signal was due to lysosomal biogenesis, we investigated the number of lysosomes and amount of a lysosomal enzyme following treatment with the two compounds. The results showed significant increases in LAMP1-positive structures and cathepsin D protein with treatment (Fig. 4C,D), indicating that inhibition of CDKs induces lysosomal biogenesis.

To determine whether TFEB is involved in the lysosomal biogenesis induced by inhibition of CDKs, we observed the nuclear localisation of TFEB following treatment with kenpaullone and purvalanol A by microscopy. Unexpectedly, kenpaullone and purvalanol A did not increase the nuclear import of TFEB, while the mTOR inhibitor Torin1 markedly increased the nuclear localisation of TFEB-sfGFP (Fig. 4E). Furthermore, treatment with kenpaullone or purvalanol A did not induce dephosphorylation of 4E-BP, a substrate of mTOR (Fig. 4F)^28^. These data indicate that the increase in lysosomal biogenesis induced by treatment with kenpaullone or purvalanol A is independent of TFEB.

**Figure 5:**
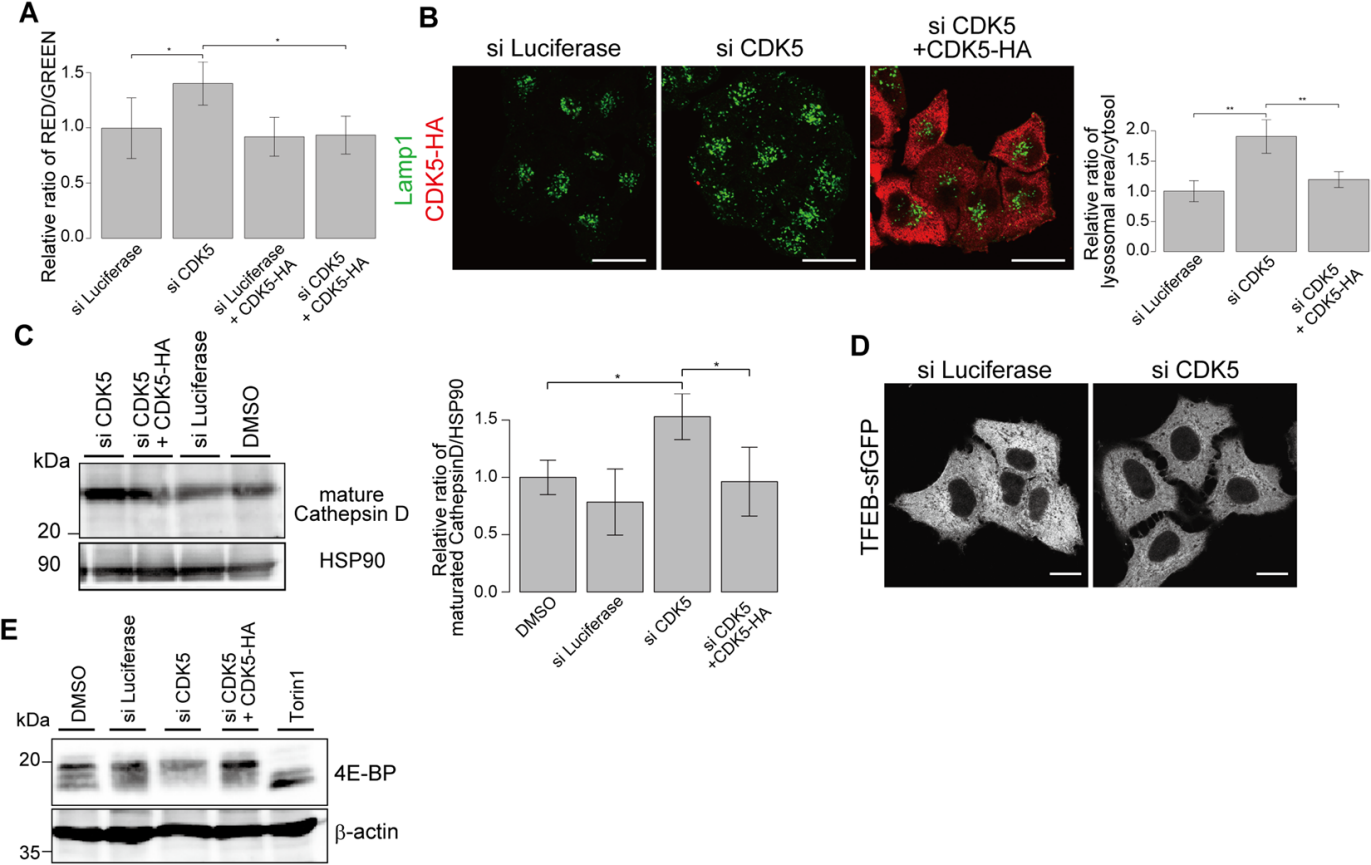
Inhibition of CDK5 promotes the lysosomal biogenesis pathway. (**A**) Knockdown of CDK5 increases the red/green ratio. HeLa cells expressing the lysosomal-METRIQ probe and rescue HeLa cells expressing siRNA-resistant CDK5-HA were cultured with Dox for 48 h and treated with siRNA targeting endogenous CDK5 or luciferase (negative control). The red/green fluorescence ratios are shown (n = 3). The data represent the mean ± SD. *p < 0.05. (**B**) Knockdown of CDK5 increases lysosomal structures. HeLa cells and siRNA-resistant CDK5-HA-expressing HeLa cells were treated with siRNA targeting endogenous CDK5 or luciferase before fixation. Cells were stained with antibodies against LAMP1 and analysed by immunofluorescence microscopy. Scale bar, 20 μm. The graph shows relative ratios of LAMP1-positive areas to cytoplasmic areas in the cells treated with the indicated siRNA. The data represent the mean ± SD; **p < 0.01. (**C**) Endogenous mature cathepsin D in HeLa cells and CDK5-HA-expressing rescue HeLa cells treated with siRNA targeting endogenous CDK5 or luciferase were analysed by immunoblotting using antibodies against cathepsin D and Hsp90. The mature cathepsin D expression levels were normalised to the HSP90 expression levels (right panel). The data represent the mean ± SD. *p < 0.05. (**D**) Inhibition of CDK5 does not induce nuclear localisation of TFEB. HeLa cells stably expressing TFEB-sfGFP were treated with siRNA targeting CDK5 or luciferase before fixation, and the cells were analysed by immunofluorescence microscopy. Scale bar, 20 μm. (**E**) Inhibition of CDK5 does not decrease mTOR activity. Endogenous phosphorylated 4E-BP in HeLa cells and CDK5-HA-expressing rescue HeLa cells treated with siRNA targeting endogenous CDK5 or luciferase were analysed by immunoblotting using antibodies against 4E-BP and β-actin (loading control).

To identify the CDK that regulates lysosomal biogenesis, we used RNA interference (RNAi) to knockdown individual CDKs targeted by kenpaullone or purvalanol A (i.e., CDK1, CDK2, CDK4 and CDK5). Analysis using the lysosomal-METRIQ probe also showed high R/G ratios with knockdown of CDK1, CDK4, and CDK5 (Figs 5A, S3A). Knockdown of CDK1, CDK4, and CDK5 but not CDK2 increased LAMP1-positive structures (Figs 5B, S3B). However, rescue of CDK1 and CDK4 knockdown by co-expression of siRNA-resistant forms of these CDKs did not restore the R/G ratios (Fig. S3A,B). Conversely, co-expression of siRNA-resistant CDK5-HA restored the R/G ratio of the lysosomal-METRIQ probe and the increase in the number of LAMP1-positive structures and cathepsin D protein expression induced by siRNA targeting CDK5 (Fig. 5A–C). Additionally, upon knockdown of CDK5, neither nuclear localisation of TFEB nor dephosphorylation of 4E-BP was promoted (Fig. 5D,E). To further characterise the effect of CDK5 on lysosomal activity, we quantified the activity of lysosomal acid phosphatase. Acid phosphatase activity tended to increase with CDK5 knockdown (Fig. S3C). We also performed quantitative PCR to investigate whether the lysosomal biogenesis caused by CDK5 knockdown is transcriptionally mediated. CDK5 knockdown upregulated cathepsin D mRNA levels, and this effect was restored by the expression of exogenous CDK5 (Fig. S3D). However, LAMP1 mRNA levels were not significantly altered upon CDK5 knockdown, indicating that CDK5 might repress some lysosomal functions to an extent sufficient for attenuation of lysosomal activity. Taken together, these results suggest that CDK5 acts as a cytosolic kinase in a lysosomal homeostasis pathway in a manner independent of TFEB and cell cycle arrest.

## Discussion

Plasmid-based ratiometric fluorescent assays are established procedures for investigation of autophagy-mediated lysosomal degradation^29,30^. In this study, we developed a novel lysosomal-METRIQ probe, DNase IIα-sfGFP-T2A-mCherry, that can be used to easily and quantitatively measure lysosomal activity through calculation of RFP/GFP ratio using a cytometer. Newly synthesised lysosomal-sfGFP is transported from the ER to lysosomes via the secretion pathway and is continuously degraded by lysosomal proteases in intact cells. Therefore, increases in the lysosomal-sfGFP signals imply impairment in membrane trafficking or lysosomal activities. In contrast, decreases in the lysosomal-sfGFP signals indicate increases in lysosomal biogenesis. The probe co-encodes cytosolic mCherry as an internal control that is stable in the cytoplasm/nucleosol. This feature enables measurement of lysosomal activity by calculation of the R/G fluorescence ratio independently of transcriptional and translational perturbations or differential expression levels between cell lines.

Fluorescently labelled bovine serum albumin (BSA; e.g., DQ Red BSA or Magic Red) is another available fluorescent marker of lysosomal activity that detects lysosomal protease activity following incorporation by endocytosis^31^. Magic Red can be used with live imaging combined with short-term fluorescence recovery after photobleaching (FRAP)^19^. Although the efficiency with which a fluorescent reagent is incorporated into a cell is dependent on the total cell number and cell density, cell number and density do not affect the R/G fluorescence ratio calculated with our developed lysosomal-METRIQ probe.

The lysosomal-METRIQ probe can be used to compare lysosomal activity among different cell lines. Based on our results, HEK293FT and H1299 cells showed lower lysosomal activity than HeLa cells, while U2OS cells showed higher activity (Fig. 1D). These data indicate that there are differences in lysosomal pathways or lysosomal activities at basal conditions. One caution with the lysosomal-METRIQ probe is that overexpression of DNase IIα-sfGFP reduces the sensitivity of the probe. When the lysosomal-METRIQ probe was driven by the CMV promoter, the degradation rate of the DNase IIα-sfGFP protein was significantly impaired, even under non-stressed conditions (data not shown). Therefore, we used a Tet-On promoter and a stably expressing cell line in this study.

Through a compound screening approach, we identified several candidates as lysosomal downregulators, including proteasome inhibitors (MG-132, lactacystin, and bortezomib) and histone deacetylase (HDAC) inhibitors (scriptaid, trichostatin A, and vorinostat) (Fig. 3). The proteasome inhibitors exhibit different structures, and treatment with these inhibitors did not increase the sfGFP signal in lysosomes (data not shown). The translocation of secretory proteins by signal peptides is not 100% efficient, and mislocalised secretory proteins in the cytoplasm are immediately degraded by the ubiquitin-proteasome system^32^. The DNase IIα-sfGFP protein in the lysosomal-METRIQ probe contains a signal peptide. Proteasome inhibition may have resulted in the accumulation of mislocalised DNase IIα-sfGFP in the cytoplasm. Alternatively, ubiquitin-mediated degradation may regulate the membrane trafficking pathway. In addition, none of the HDAC inhibitors increased the sfGFP signal in lysosomes (data not shown). HDAC inhibitors remove the acetyl groups from histones, leading to the formation of condensed and transcriptionally silenced chromatin. The HDAC-dependent condensed chromatin may contain lysosomal- or membrane trafficking-related genes. Alternatively, hydroxamate-based HDAC inhibitors, including scriptaid, trichostatin A, and vorinostat, might interfere with a lysosomal membrane trafficking pathway or multiple pathways independent of HDAC inhibition.

We also identified dozens of compounds that upregulate lysosomal activity. Among them, the inhibitors jervine and cyclopamine target the membrane protein Smoothened, which functions in hedgehog signalling, and treatment with these compounds increased lysosomal activity (Fig. 3 and Table S1). However, vismodegib, another Smoothened inhibitor in the library, did not affect lysosomal activity at all (Table S1). The library also includes AY 9944, which inhibits hedgehog signalling via different mechanisms and showed the opposite effect on lysosomal activity (Table S1). In terms of structure, jervine and cyclopamine are steroidal alkaloids, while vismodegib and AY 9944 have different structures. Knockdown of Smoothened by siRNA did not change the activity of the lysosomal-METRIQ probe (Fig. S2G). As cholesterol is abundant in the lysosomal membrane, treatment with steroidal alkaloids might activate the lysosomal pathway by changing the membrane composition of lysosomes independently of hedgehog signalling.

The major compounds identified in the screening as activators of the lysosomal pathway were CDK inhibitors including purvalanol A and kenpaullone. We confirmed that these compounds not only induced activation of the lysosomal-METRIQ probe but also increased the levels of cathepsin D and the number of lysosomes (Fig. 4C,D). Although these two compounds are structurally different, both potentially inhibit CDK1, CDK2, CDK4, and CDK5^26,27^. Indeed, treatment with these compounds induces cell cycle arrest at any cycle stage^33,34^. We hypothesised that organelle growth continued even after cell cycle arrest caused by the inhibitor and resulted in increased numbers of lysosomes. In such a case, cell cycle arrest by other stimuli should also increase the number of lysosomes. However, induction of DNA damage with HU or etoposide, or microtubule depolymerisation by nocodazole, which cause arrest at the G1/S, G2, or M phase, respectively, did not upregulate the lysosomal pathway (Fig. 4B). Although the mechanism by which organelle size is coordinated remains unclear^35^, these data indicate that cell cycle arrest might suppress organelle growth, and that activation of the lysosomal pathway by CDK inhibition is independent of cell cycle arrest.

We found that knockdown of CDK5 leads to lysosomal biogenesis at least partially through transcription, independent of the nuclear translocation of TFEB (Figs 5, S3). The substrate or substrates of CDK5 involved in lysosomal biogenesis remains unknown. Despite its sequence similarities with CDK1, CDK5 is an atypical CDK and does not participate in cell cycle progression in proliferating cells^36,37^. CDK5 has emerged as an important regulator of neuronal migration. However, accumulating evidence has revealed multifunctional roles of CDK5 in neuronal and non-neuronal cells. CDK5 phosphorylates a diverse list of substrates that function in controlling the organisation of membrane dynamics, signalling cascades, the cytoskeleton and focal adhesions. Our data indicate that one or more transcription factors might be phosphorylated by CDK5 to suppress lysosomal activity. However, CDK5 also modulates endocytosis at synapses by regulating the phosphorylation of dynamin I and amphiphysin I^38,39^. Furthermore, CDK5 phosphorylates AATYK1A to regulate early and recycling endosomes^40,41^. Alternatively, a recent report showed that Pho85, a yeast homologue of CDK5, phosphorylates PI3P-5 kinase and activates phosphatidylinositol 3,5-bisphosphate (PI(3,5)P_2_) production to protect vacuoles from hyperosmotic stress^42^. Interestingly, *pho85*Δ yeast cells exhibit enlarged vacuoles without loss of vacuolar protease activity—or, rather, increased autophagic degradative activity^42,43^. These reports imply that CDK5 might regulate lysosomal biogenesis post-translationally through membrane trafficking. Collectively, these data suggest that CDK5 maintains lysosomal homeostasis by affecting lysosomal biogenesis. Future work on the regulatory roles of CDK5/PHO85 could reveal a mechanism that modulates lysosome populations.

## Materials and Methods

### Cell culture

HeLa, HEK293FT, H1299, HT1080, and U2OS cells were cultured in Dulbecco’s modified Eagle’s medium (Nacalai Tesque, Kyoto, Japan) supplemented with 10% fetal bovine serum (FBS; Biosera, Ringmer, UK) and, 50 mg/ml penicillin and streptomycin (regular medium) in a 5% CO_2_ incubator. Tetracycline-On (Tet-On) cells were generated by lentiviral transduction with a pCW57.1 vector containing the single-vector Tet-On component. For drug treatment, cells were incubated for the indicated times in medium containing one or a cocktail of the following reagents: 0.2 μM bafilomycin A_1_ (LC Laboratories, Woburn, MA, USA), 50 μg/ml brefeldin A (Wako, Osaka, Japan), 1 μM Torin1 (Tocris Bioscience, Ellisville, MO, USA), 10 μg/ml E64d (Peptide Institute, Osaka, Japan), 100 μM pepstatin A (Peptide Institute), 20 μg/ml leupeptin (Peptide Institute), 10 μM kenpaullone (Cayman Chemical, Ann Arbor, MI, USA), 10 μM purvalanol A (Cayman Chemical), 10 μM jervine (Wako), 10 μM BMS-345541 (Cayman Chemical), 2.5 mM HU (Wako), 20 μM etoposide (Cayman Chemical), 5 μg/ml nocodazole (Cayman Chemical), or 1 μg/ml doxycycline (Clontech, Mountain View, CA, USA).

### Plasmids

To generate lysosomal-METRIQ probes, DNase IIα, ASAH, and LAMP1 cDNA was amplified from HEK293FT total cDNA and inserted into a pCW57.1 vector (Addgene plasmid #41393) along with sfGFP, T2A peptide, and mCherry. For pMRX-IP TFEB-sfGFP, TFEB cDNA amplified from HEK293FT total cDNA was inserted into a pMRX-IP^44^ vector together with sfGFP. CDK family cDNA was amplified from mouse embryonic fibroblast cDNA and inserted into pMRX-IB vector with a human influenza hemagglutinin (HA) tag. Since mouse CDK genes contain several base substitutions, they are considered resistant to siRNA targeting endogenous human CDK genes. Flag-tagged IKKβ and its mutant were amplified from pCR-Flag-IKKβ (Addgene plasmid #15465) or pCR-Flag-IKKβ-KM (Addgene plasmid #15466) plasmids and inserted into pMRX-IB vector. The following plasmids were generated: pCW DNaseIIα-sfGFP-T2A-mCherry, pCW ASAH-sfGFP-T2A-mCherry, pCW LAMP1-sfGFP-T2A-mCherry, pMRX-IP TFEB-sfGFP, pMRX-IB CDK1-HA, pMRX-IB CDK4-HA, pMRX-IB CDK5-HA, pMRX-IB Flag-IKKβ, and pMRX-IB Flag-IKKβ (K44A). pCMV-VSVG (Addgene plasmid #8454) and psPAX2 (Addgene plasmid #12260) were used for lentivirus production. pCMV-VSVG and Gag were used for retrovirus production.

### Antibodies

Rabbit polyclonal anti-LAMP1 antibodies were gifted from Y. Tanaka (Kyushu University, Fukuoka, Japan). Rabbit polyclonal anti-Trapα and anti-TMP21 antibodies were gifted from R. S. Hegde (MRC LMB). Rabbit polyclonal anti-TFEB (#4240), rabbit polyclonal anti-4E-BP1 (#9644), and rabbit polyclonal anti-IKKβ (#8943) antibodies were purchased from Cell Signaling Technology (Danvers, MA, USA). Mouse monoclonal anti-Flag-tag (Clone No. 1E6) and mouse monoclonal anti-β-actin (Clone No. 2F3, Cat No. 013-24553) antibodies were purchased from Wako. Mouse monoclonal anti-HSP90 (610419) antibodies were purchased from BD Bioscience (Tokyo, Japan). Goat polyclonal anti-Cathepsin D (AF1014) antibodies were purchased from R&D Systems (Minneapolis, MN, USA). Mouse monoclonal anti-GM130 and rabbit polyclonal anti-EEA1 antibodies were purchased from MBL (Tokyo, Japan).

### Screening of drugs

A drug library, the SCADS Inhibitor Kit, was provided by the Screening Committee of Anticancer Drugs supported by a Grant-in-Aid for Scientific Research on Innovative Areas, Scientific Support Programs for Cancer Research, from The Ministry of Education, Culture, Sports, Science and Technology, Japan. HeLa Tet-On cells harbouring the lysosomal-METRIQ probe were cultured in regular medium with 1 μg/ml Dox for 48 h and treated with the compound of library at a concentration of 10 μM for 12 h. The R/G fluorescence ratio was measured using a flow cytometer. Compounds with an R/G ratio below 0.75 were defined as downregulator candidates of the lysosomal pathway, and are shown in blue. Compounds with an R/G ratio above 1.25 were defined as upregulator candidates of lysosomal biogenesis, and are shown in red. Drugs for which the population of living cells was reduced to half or less than half of the control population as well as compounds with high auto-fluorescence were excluded.

### siRNA knockdown experiments

Stealth RNAi oligonucleotides (Thermo Fisher Scientific, Lafayette, CO, USA) and Dicer-substrate siRNA (DsiRNA) oligonucleotides (Integrated DNA Technologies, Coralville, IA, USA) were used for siRNA experiments. The sequences used included the following: a luciferase stealth RNAi sequence (5′-CGCGGUCGGUAAAGUUGUUCCAUUU-3′), a human Smoothened stealth RNAi sequence (5′-UGACCUCAAUGAGCCCUCAGCUGAU-3′), human IKKβ stealth RNAi sequences (#1: 5′-CCGCCAUGAUGAAUCUCCUCCGAAA-3′; #2: 5′-GCAGAAGAGUGAGGUGGACAUUGUU-3′), a luciferase DsiRNA sequence (5′-ACUGAGACUACAUCAGCUAUUCUGA-3′), a human CDK1 DsiRNA sequence (5′-GUACUGCAAUUCGGGAAAUUUCUCT-3′), a human CDK2 DsiRNA sequence (5′-GGUUUUGUAAUGACAGUGCUAAAAA-3′), a human CDK4 DsiRNA sequence (5′-UGACUGACAAAGCUUAGAAAGGAAC-3′), and a human CDK5 DsiRNA sequence (5′-ACUUUGGUUUUUGAAUUCUGUGACC-3′). HeLa cells were seeded onto a 12-well plate at 50% confluency. siRNA transfection was performed with Lipofectamine RNAiMAX transfection reagent (Thermo Fisher Scientific) using 20 nM siRNA in Opti-MEM (Gibco, Tokyo, Japan). For CDK knockdown, cells were incubated for 2 days before analysis. For knockdown of IKKβ and Smoothened, at 2 days post transfection, the cells were again transfected with the same siRNA and cultured for an additional 3 days.

### Lentiviral and retroviral infection and of stable cell line generation

Stable cell lines were generated using a lentiviral or retroviral expression system. HEK293FT cells were transiently co-transfected with lentiviral or retroviral vectors using PEI MAX reagent (Polysciences, Warrington, PA, USA). After culturing for 72 h, growth medium containing the lentivirus or retrovirus was collected. HeLa cells were incubated with the collected virus-containing medium with 10 mg/ml polybrene for 48 h. Uninfected cells were removed using 1 μg/ml puromycin (InvivoGen, San Diego, CA, USA) or 5 μg/ml blasticidin S (Wako).

### Fluorescence microscopy

Cells were plated on coverslips, fixed in 3.7% formaldehyde in phosphate-buffered saline (PBS) for 15 min, and observed under a confocal laser microscope (FV1000 IX81: Olympus, Tokyo, Japan) using a 100× oil immersion objective lens with a numerical aperture (NA) of 1.40. For immunostaining, fixed cells were permeabilised with 50 μg/ml digitonin in PBS for 5 min, blocked with 10% FBS or newborn bovine serum in PBS for 45 min, and incubated with primary antibodies for 1 h. After washing, the cells were incubated with Alexa Fluor 488- or 647-conjugated goat anti-rabbit or goat anti-mouse IgG secondary antibodies (Thermo Fisher Scientific) for 1 h.

### Flow cytometry

Cells were trypsinised with EDTA and recovered by detachment from the dish. The cells were passed through a 70-μm cell-strainer, and resuspended in 10% FBS and 1 μg/ml 4′,6-diamidino-2-phenylindole (DAPI) in PBS for flow cytometry analysis using a CytoFLEX S flow cytometer equipped with NUV 375 nm (DAPI), 488 nm (GFP), and 561 nm (mCherry) lasers (Beckman Coulter, Brea, CA, USA). Dead cells were detected by DAPI staining. In each sample, 10,000 cells were acquired and R/G fluorescence ratios were calculated as red fluorescence intensity divided by the green fluorescence intensity in mCherry-positive cells.

### Immunoblotting

Cells were washed with cold PBS and lysed in lysis buffer (1% Triton X-100, 50 mM Tris/HCl pH 7.5, 1 mM EDTA, and 150 mM NaCl) or RIPA buffer (0.5% Triton X-100, 10 mM Tris/HCl pH 8.0, 1 mM EDTA, 0.5 mM EGTA, 0.1% sodium dodecyl sulphate [SDS], 0.02% sodium deoxycholate, and 140 mM NaCl) for TFEB blotting, supplemented with protease-inhibitor cocktail (EDTA-free) (Nacalai Tesque), 1 mM phenylmethanesulphonylfluoride, and phosphatase inhibitors (5 mM NaF, 5 mM NaN_3_, 5 mM 4-nitrophenylphosphate, 5 mM Na_2_P_2_O_4_, and 5 mM β-glycerophosphate) for 15 min at 4 °C. The lysates were clarified by centrifugation at 20,630 × *g* for 5 min and 6× SDS sample buffer was added. The samples were boiled at 95 °C for 5 min before SDS/polyacrylamide gel electrophoresis (SDS/PAGE). Twenty micrograms of protein per lane was separated by SDS/PAGE and transferred to a polyvinylidene difluoride membrane (Millipore, Billerica, MA, USA). Immunoblot analysis was performed with the indicated antibodies and the immunoreactive proteins were visualised using ImmunoStar Zeta (Wako).

### Acid phosphatase assay

The acid phosphatase activity of lysosomes was measured using an Acid Phosphatase Assay Kit (Colorimetric) according to the manufacturer’s instructions (Cat No. ab83367, Abcam, Cambridge, UK). The acid phosphatase activity was normalised to the protein concentration.

### RNA extraction, reverse transcription and quantitative PCR

Total RNA was extracted from cells using ISOGEN II (NIPPON GENE, Tokyo, Japan). Reverse transcription was performed using ReverTra Ace reverse transcription reagents (TOYOBO LIFE SCIENCE, Osaka, Japan). The gene-specific primers were as follows: human Lamp1, 5′-GCGTACCTTTCCAACAGCAG-3′ (forward) and 5′-GCCGCTCACGTTGTACTTGT-3′ (reverse); human Cathepsin D, 5′-GACATCCACTATGGCTCGGG-3′ (forward) and 5′-TGCCTCTCCACTTTGACACC-3′ (reverse); and human GAPDH, 5′-CCACATCGCTCAGACACCA-3′ (forward) and 5′-GGCAACAATATCCACTTTACCAGAG-3′ (reverse). Relative quantification of gene expression was performed according to the 2 (−ΔΔCT) method. The housekeeping gene GAPDH was used as an internal control to normalise the variability in expression levels.

## Supplementary information

Supplemental Figures

Table S1
