# Supplementary material for: Identification of a factor controlling lysosomal homeostasis using a novel lysosomal trafficking probe
Source: Sci Rep. 2019 Aug 12;9:11635. doi: 10.1038/s41598-019-48131-2 (PMC6690932; doi:10.1038/s41598-019-48131-2)
Supplement: Supplementary file 1 — Supplemental Figures [file 41598_2019_48131_MOESM1_ESM.pdf]

Identification of a factor controlling lysosomal homeostasis using a novel lysosomal trafficking probe

Shunsuke Ishii[1], Akira Matsuura[2], Eisuke Itakura[2*]

[1]Department of Biology, Graduate school of Science and Engineering, Chiba University, Inage-ku, Chiba 263-8555, Japan.

[2]Department of Biology, Graduate school of Science, Chiba University, Inage-ku, Chiba 263-8555, Japan.

* Corresponding author: eitakura@chiba-u.jp (EI)

Supplementary Information Ishii et. al.,

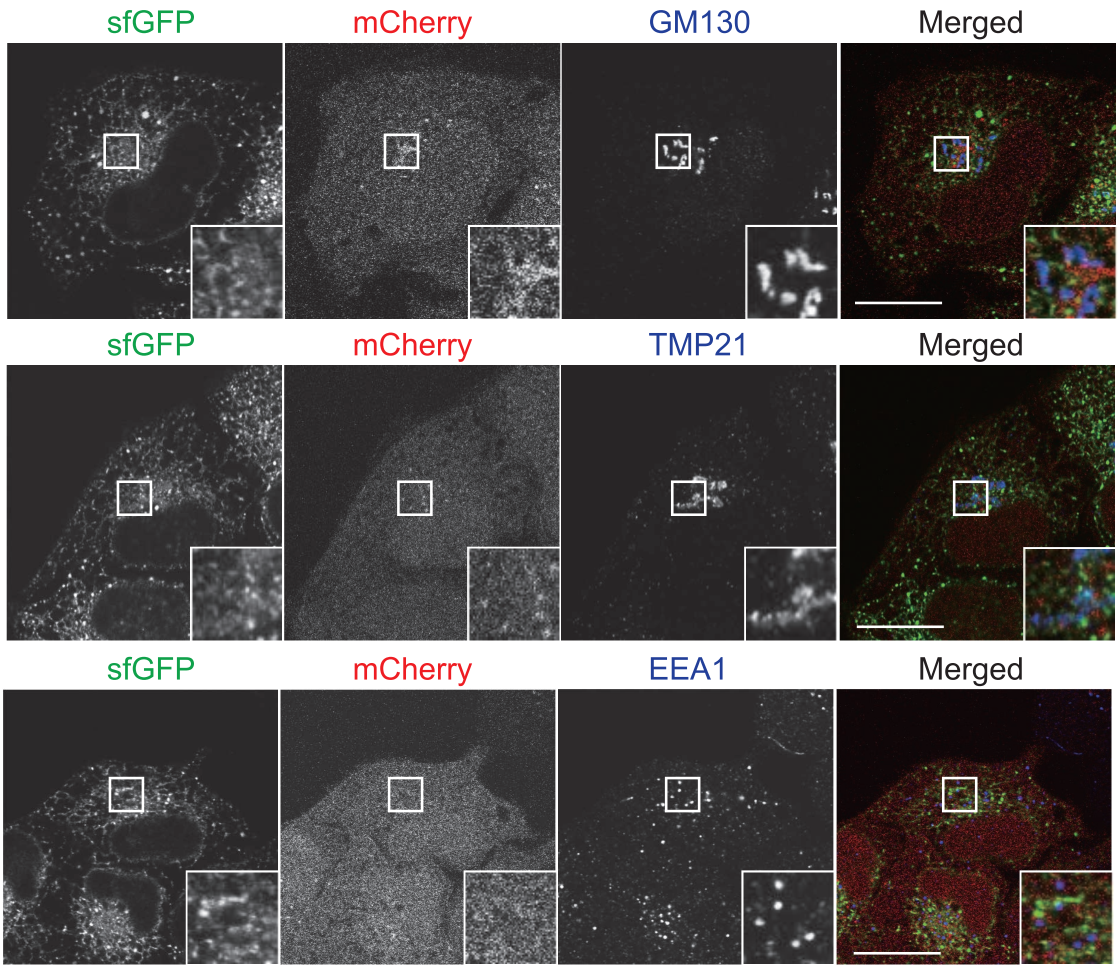

Supplemental Figure 1. DNase II α -sfGFP does not co-localise with the Golgi and early endosome markers. HeLa cells expressing the lysosomal -METRIQ probe were incubated in medium containing Dox for 48 h before fixation. The c ells were stained with antibodies against GM130, TMP21, and EEA1 and analysed by immunofluorescence microscopy. A magnified image of the indicated region is shown in the inset. Scale bar, 20 µm

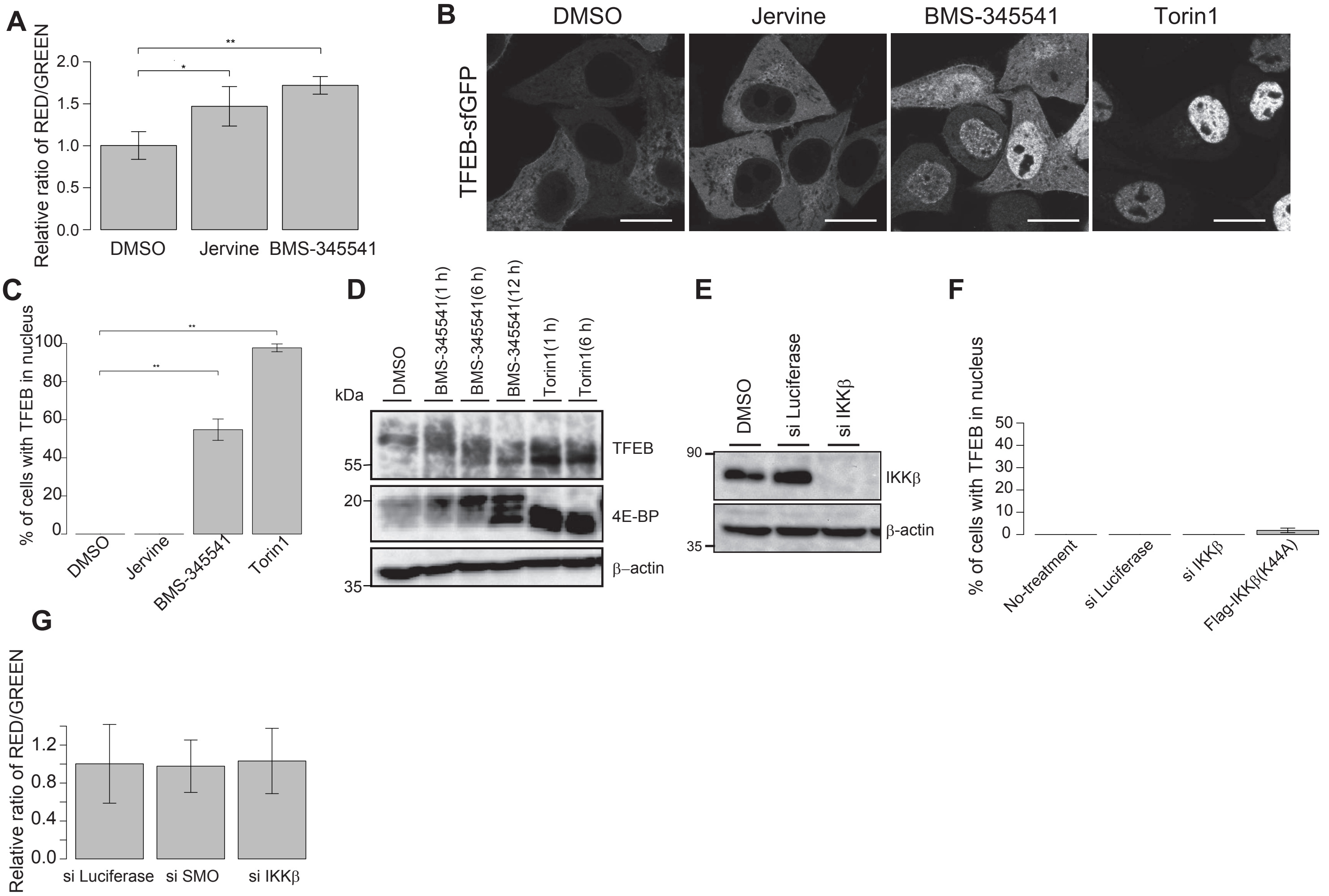
A
Relative ratio of RED/GREEN
2.0
1.5
1.0
0.5
0.0
**
*
DMSO
Jervine
BMS-345541
B
DMSO
Jervine
BMS-345541
Torin1
TFEB-sfGFP
C
% of cells with TFEB in nucleus
100
80
60
40
20
0
**
**
DMSO
Jervine
BMS-345541
Torin1
D
kDa
DMSO
BMS-345541(1 h)
BMS-345541(6 h)
BMS-345541(12 h)
Torin1(1 h)
Torin1(6 h)
55
20
35
TFEB
4E-BP
β–actin
E
DMSO
si Luciferase
si IKKβ
90
35
IKKβ
β-actin
F
% of cells with TFEB in nucleus
50
40
30
20
10
0
No-treatment
si Luciferase
si IKKβ
Flag-IKKβ(K44A)
G
Relative ratio of RED/GREEN
1.2
0.8
0.4
0.0
si Luciferase
si SMO
si IKKβ

Supplemental Figure 2. Involvement of IKKβ and hedgehog signalling inhibitors in lysosomal function. (A) Smoothened or IKKβ inhibitors increase lysosomal activity. HeLa cells expressing the lysosomal-METRIQ probe were cultured in medium with Dox for 48 h and treated with vehicle (DMSO), jervine, or BMS-345541 for 12 h prior to flow cytometry. The red/green fluorescence ratios are shown. (B) Nuclear translocation of TFEB by IKKβ inhibitor. HeLa cells stably expressing TFEB-sfGFP were treated with vehicle (DMSO), jervine, or BMS-345541 for 12 h before fixation and were analysed by microscopy. Scale bar, 20 μm. (C) The percentage of cells with TFEB in the nucleus is shown (n = 3). The data represent the mean ± SD. The data are representative of at least three independent experiments and > 100 cells were counted; ** p <0.01. (D) Treatment with BMS-345541 decreases the phosphorylation levels of TFEB in a time dependent manner independently of mTOR inhibition. HeLa cells treated with BMS-345541 or Torin1 for 1, 6, or 12 h were analysed by immunoblotting using antibodies against TFEB, 4E-BP and β-actin (loading control). (E) Treatment of cells with siRNA against IKKβ significantly reduces the levels of endogenous IKKβ. HeLa cells were treated with siRNA targeting IKKβ and were analysed by immunoblotting using antibodies against IKKβ and β-actin (loading control). (F) Inhibition of IKKβ does not induce nuclear localisation of TFEB. HeLa cells stably expressing TFEB-sfGFP were treated with siRNA targeting IKKβ, or transfected with plasmid encoding IKKβ K44A. The percentage of cells with TFEB in the nucleus is shown (n = 3). The data represent the mean ± SD. The data are representative of at least three independent experiments and > 100 cells were counted. (G) Knockdown of IKKβ or Smoothened does not affect lysosomal activity. HeLa Tet-On cells expressing the lysosomal-METRIQ probe were treated with siRNA targeting Smoothened or IKKβ, and cultured in medium with Dox for 48 h prior to analysis. The red/green fluorescence ratios are shown (n = 4). The data represent the mean ± SD.

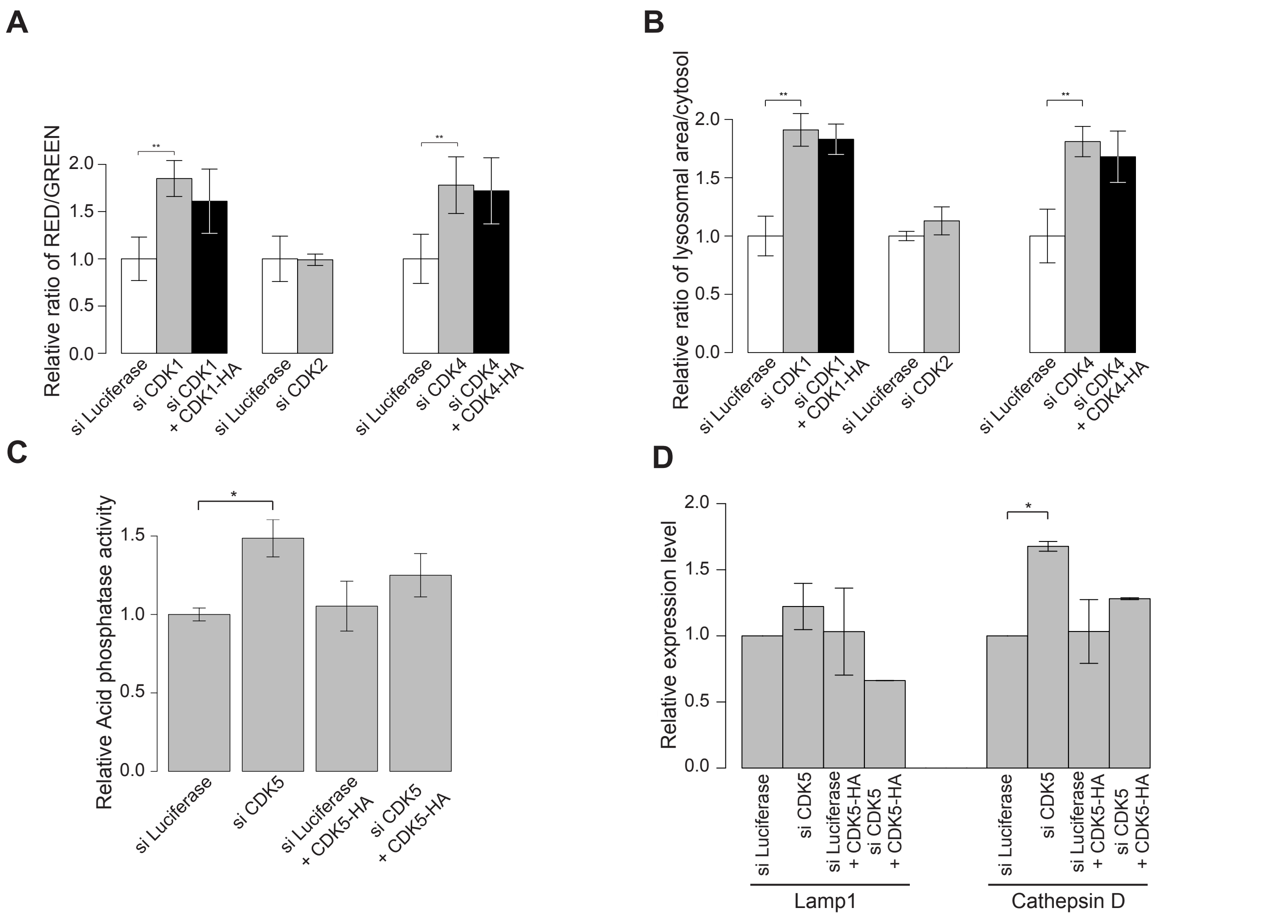

Supplemental Figure 3. **Involvement of CDK in lysosomal function.** (A) The red/green ratio is increased by knockdown of CDK1 or CDK4 butis not rescued. HeLa cells and CDK1-HA- or CDK4-HA-expressing rescue HeLa cells expressing the lysosomal -METRIQ probe were cultured with Dox for 48 h and treated with the indicated siRNAs. The red/green fluorescence ratios are shown (n = 3). The data represent the mean ± SD; **p < 0.01. (B) Increased lysosom al structures by knockdown of CDK1 or CDK4 are not rescued by overexpression of siRNA - resistant mutated CDK1 -HA or CDK4 -HA. The graph shows the relative ratios of LAMP1 -positive area s to cytoplasmic areas in the cells treated with the indicated siRNAs. The data represent the mean ± SD; **p < 0.01. (C) Analysis of lysosomal activity under CDK5 knockdown. HeLa cells and rescue HeLa cells expressing siRNA-resistant CDK5-HA were treated with siRNA targeting endogenous CDK5 or luciferase (negative control). Acid phosphatase activity was measured for each cell lysate using an Acid Phosphatase Assay Kit. The relative acid phosphatase activities are shown (Normalised to that of cells treated with siRNA against Luciferase) (n = 3). The data represent the mean ± SD. *p < 0.05. (D) Upregulation of cathepsin D mRNA levels by CDK5 knockdown. cDNA was synthesized from HeLa cells and rescue d HeLa cells treated with siRNA targeting CDK5 or luciferase. Quantitative PCR wa s performed for endogenous Lamp1 and c athepsin D. The r elative expression levels of the indicated genes are shown (n = 3). The data represent the mean ± SE; *p < 0.05.

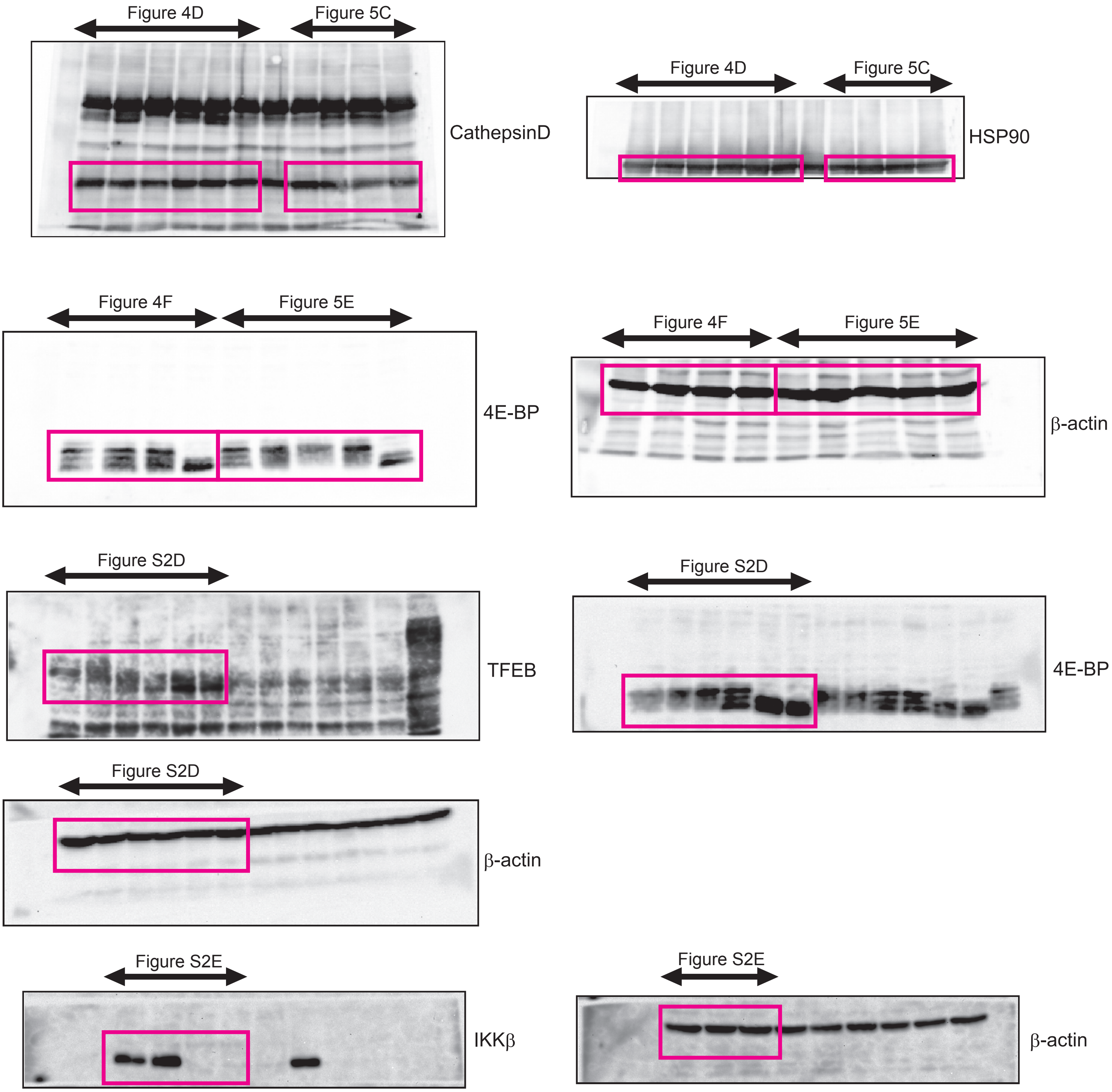

Supplemental Figure 4. **Full Length blots of CathepsinD, 4E-BP, TFEB, IKKβ, HSP90 and β-actin (Figure 4, 5, S1)**
